# Synthesis and characterization of Mg–Zn ferrite based flexible microwave composites and its application as SNG metamaterial

**DOI:** 10.1038/s41598-021-87100-6

**Published:** 2021-04-07

**Authors:** Md Atiqur Rahman, Mohammad Tariqul Islam, Mandeep Singh Jit Singh, Md Samsuzzaman, Muhammad E. H. Chowdhury

**Affiliations:** 1 Department of Electrical, Electronic and Systems Engineering, Faculty of Engineering and Built Environment, Universiti Kebangsaan Malaysia, 43600 Bangi, Selangor Malaysia; 2 Department of Computer and Communication Engineering, Faculty of Computer Science and Engineering, Patuakhali Science and Technology University, Dhaka, Bangladesh; 3 Department of Electrical Engineering, Qatar University, 2713, Doha, Qatar

**Keywords:** Engineering, Electrical and electronic engineering, Materials science, Soft materials

## Abstract

In this article, we propose SNG (single negative) metamaterial fabricated on Mg–Zn ferrite-based flexible microwave composites. Firstly, the flexible composites are synthesized by the sol-gel method having four different molecular compositions of Mg_x_Zn_(1−x)_Fe_2_O_4,_ which are denoted as Mg_20_, Mg_40,_ Mg_60,_ and Mg_80_. The structural, morphological, and microwave properties of the synthesized flexible composites are analyzed using X-ray diffraction (XRD), field emission scanning electron microscopy (FESEM), and conventional dielectric assessment kit (DAK) to justify their possible application as dielectric substrate at microwave frequency regime. Thus the average grain size is found from 20 to 24 nm, and the dielectric constants are 6.01, 5.10, 4.19, and 3.28, as well as loss tangents, are 0.002, 0.004, 0.006, and 0.008 for the prepared Mg–Zn ferrites, i.e., Mg_20,_ Mg_40,_ Mg_60,_ and Mg_80_ respectively. Besides, the prepared low-cost Mg–Zn ferrite composites exhibit high flexibility and lightweight, which makes them a potential candidate as a metamaterial substrate. Furthermore, a single negative (SNG) metamaterial unit cell is fabricated on the prepared, flexible microwave composites, and their essential electromagnetic behaviors are observed. Very good effective medium ratios (EMR) vales are obtained from 14.65 to 18.47, which ensure the compactness of the fabricated prototypes with a physical dimension of 8 × 6.5 mm^2^_._ Also, the proposed materials have shown better performances comparing with conventional FR4 and RO4533 materials, and they have covered S-, C-, X-, Ku-, and K-band of microwave frequency region. Thus, the prepared, flexible SNG metamaterials on Mg_x_Zn_(1−x)_Fe_2_O_4_ composites are suitable for microwave and flexible technologies.

## Introduction

In present-day innovation, oxide-based flexible microwave composites are pulling in enormous significance in light of their exceptional physical properties and likely applications in the territory of microwave and nanotechnology^1,2^. Due to having high electrical resistivity and nano-scaled size alongside selective physical and substance structures, among others, the metal ferrites in their two structures; spinel ferrite (as mild) and hexaferrite (as hard) is a conspicuous applicant as electromagnetic materials^3^. In spinel structure, ferrites, having high chemical and thermal dependability, offer multifunctional materials applied in different fields, for example, biomedicine, catalysis, magnetic recording, and detecting^4,5^. Metal-based composites have drawn more attention than other composites. A portion of these materials containing Mg, Zn, Fe, Ag, or Co particles arbitrarily disseminated in porous ferrite host was synthesized to acquire negative electromagnetic parameters in the radio frequency range^6–8^, and they also been broadly used in quite a lot of applications like energy harvesting^9^, space application^10,11^, filter design^12^, antenna design^13^, electromagnetic absorber^14–16^, etc. Moreover, the development of flexible composite is a highly demandable field in the microwave communication system due to having superior properties, low fabrication cost, ease of synthesis. They include various organic and inorganic materials, liquid metals, polydimethylsiloxane, and liquid crystal polymers^17–20^. To achieve such flexible ferrite composites, researchers have developed different types of metal ferrites such as MgFe_2_O_4_, CoFe_2_O_4_, ZnFe_2_O_4_, Mg_x_Zn_(1−x)_Fe_2_O_4_ for various applications like as flexible sensors, flexible battery, flexible memory, flexible displays, and others which may not possible with rigid materials^21–25^.

Among the metal ferrites, Magnesium Zinc Ferrite (MgZnFe_2_O_4_) is nothing but a combination of iron oxide (Fe_2_O_3_) and Mg–Zn metal with typical spinel assemblage. The universal formulary A_x_B_(1−x)_Fe_2_O_4_, where “A” and “B” both are divalent transition ions. The MgZnFe_2_O_4_ is fundamentally a dual oxide arrangement (MgO–ZnO–Fe_2_O_2_) that has maybe applicable as absorbents, semiconductors, and catalysts^26^. In original form, and Fe_2_O_4_ is a dielectric insulator, and MgO and ZnO are p-type semiconductors. The MgZnFe_2_O_4_ is the combined form of the MgO, ZnO, and Fe_2_O_4,_ and it offers few extraordinary electrical characteristics that do not exist at their discrete elements^27,28^. The dielectric and physical properties of MgZnFe_2_O_4_ are strongly influenced by the synthesis methods. From the time being, different methods were used by the researchers to synthesis crystalline MgZnFe_2_O_4_ like hydrothermal^29^, solid-state reaction^30^, mechano-chemical synthesis^31^, and sol–gel^32–35^. However, it has not been discovered much for microwave applications such as dielectric substrate materials for microwave antenna or metamaterials. The materials in nature are made of atoms or molecules, while the metamaterial is engineered with artificially ordered repetitive structures; each structure is known as a unit cell, like an atom of materials^36–38^. The property of a metamaterial depends on the structure of the unit cell, equivalent to the atom or molecule of natural materials. The performance of metamaterial could vary upon the modification of those unit cells^39,40^. In contrast, metamaterial may be designed to manipulate numerous properties (such as negative dielectric permittivity, negative magnetic permeability, refractive index close to zero, negative refractive index) of those applications influenced by the electromagnetic wave that cannot be found in nature^41–44^. The tunable negative permeability and permittivity of meta-composites can be accomplished by modifying their microstructures and compositions^45–49^. Typically, the metamaterials are designed on solid substrates fabricated on FR-4, Rogers, silicon, Teflon, and Taconic materials.

Thus, in this work, we at first attempted to synthesize and characterize flexible microwave composites based on Mg–Zn ferrite [Mg_x_Zn_(1−x)_Fe_2_O_4_] with four different concentrations of Mg to form SNG metamaterial.

The flexible microwave substrates are prepared using the sol-gel method to form flexible metamaterials. The sol-gel method offers numerous advantages like good stoichiometric control, high homogeneity, high productivity at low temperatures, and also able to produce unalloyed ultrafine powders. The advantages of the prepared composites are they are cost-effective, highly flexible, lightweight, and applicable incase of wearable devices. Field emission scanning electron microscopy (FESEM), X-ray diffraction (XRD), and dielectric assessment kit (DAK) was utilized to investigate the surface morphology, crystalline structures, and microwave properties of the developed Mg–Zn ferrite based flexible composites. Furthermore, metamaterial structures were fabricated on all these composites, which performed as the flexible substrates, and their transmission (S_21_) coefficients and effective parameters were analyzed using the commercially available CST Microwave Studio and MATLAB. Also, the proposed materials are compared with conventional FR4 and RO4533 materials, and better performances were observed by the proposed composites. At last, very good EMR values are found in all cases with and it is observed that the designed metamaterial on flexible Mg_x_Zn_(1−x)_Fe_2_O_4_ composites can meet all the expectations for S-, C-, X-, Ku-, and K-band applications and in the field of flexible microwave technologies.

## Experimentals

### Preparation of Mg–Zn Ferrite based flexible composites

**Figure 1:**
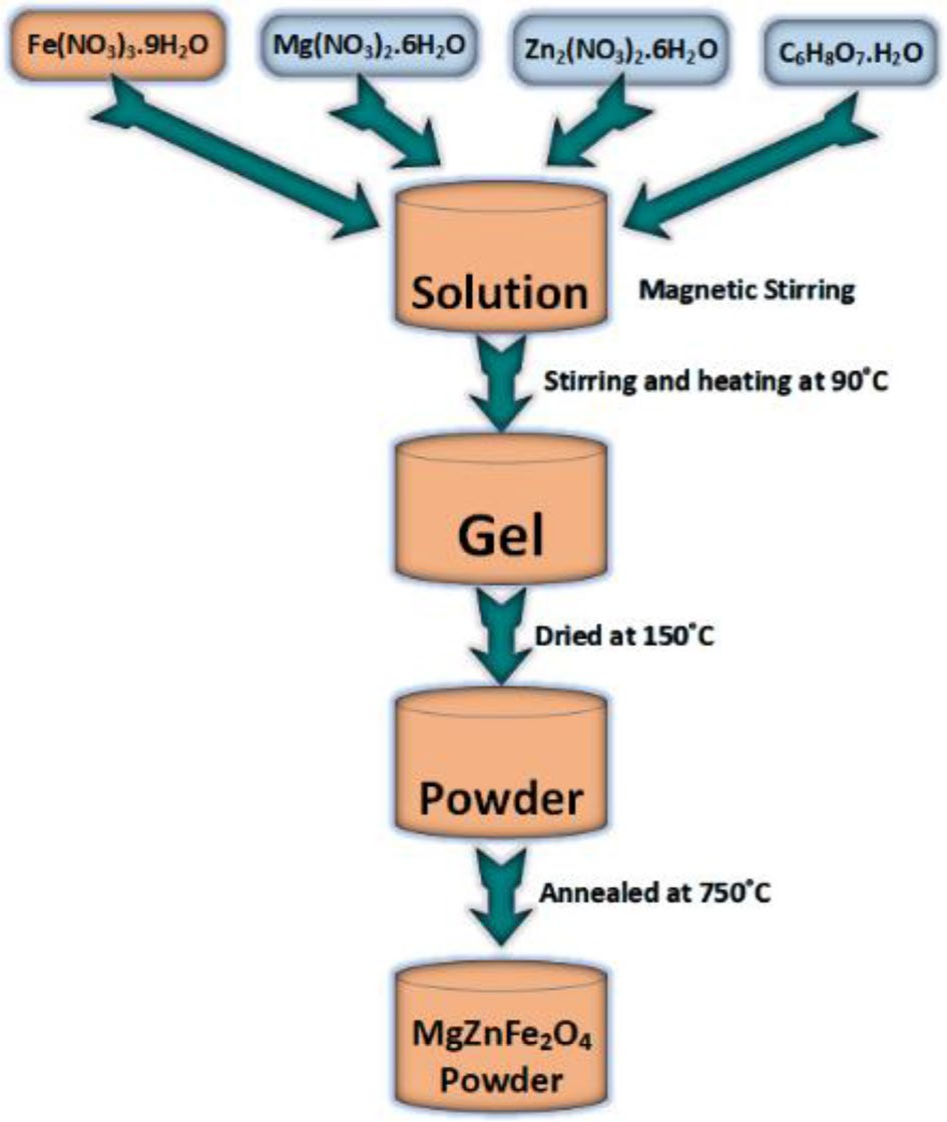
Flowchart of MgZnFe_2_O_4_ nanoparticle preparation.

The overall synthesis steps of Mg–Zn ferrite [Mg_x_Zn_(1−x)_Fe_2_O_4_] based flexible composites through the sol-gel method are illustrated in Fig. 1 with a flow chart. Magnesium nitrate hexahydrate [Mg(NO_3_)_2_∙6H_2_O], zinc nitrate [Zn_2_(NO_3_)_2_∙6H_2_O], and ferric nitrate [Fe(NO_3_)_3_∙9H_2_O] are taken as primary substances. To characterize the compositional effect of Mg and Zn content on ferrites, there are four different molar ratios taken such as 20%, 40%, 60%, and 80% of Mg nitrate and labeled as Mg_20_, Mg_40_, Mg_60_, and Mg_80_. (1) For Mg_20_, 20% of Mg nitrate and 80% of Zn nitrate is used. Similarly, (2) for Mg_40_, 40% of Mg nitrate and 60% of Zn nitrate is taken as a molar ratio. In this manner, (3) for the Mg_60_ 60% of Mg nitrate and 40% of Zn nitrate, and (4) for Mg_80_ 80% of Mg nitrate and 20% of Zn nitrate is weighted as a molar ratio. The mixers are slowly dissolved in distilled water with magnetic stirring, and also the citric acid (C_6_H_8_O_7_∙H_2_O) is added as a chelating agent, and hence, a quite transparent gelatinous solution is found having light red color. Later, the appeared solutions are stirring continuously for about 5 hours at 90 °C. Thus, a reddish gel is formed, which is further dried at 150 °C by transferring the gel into a furnace. The nanoparticle was then achieved by grinding the obtained precursor and, finally, calcined at 750 °C for one hour to complete the chemical process.

**Figure 2:**
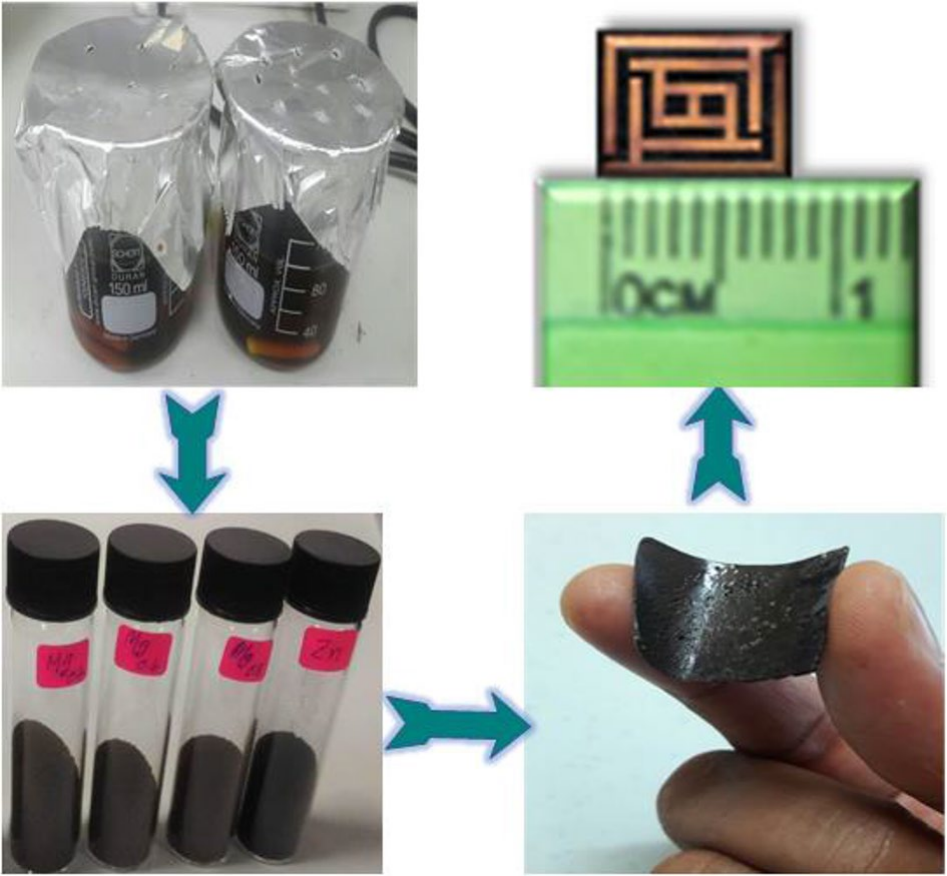
Flowchart of MgZnFe_2_O_4_ nanoparticle-based flexible metamaterial cell.

Figure 2 shows the main preparation steps of the flexible substrate-based metamaterials from their gel states to fabricated metamaterial unit cells. Firstly, the flexible substrate is prepared by adding the synthesized MgZnFe_2_O_4_ nanoparticle into the PVA glue at a ratio of 1 g powder to 10 mL of PVA glue and later dried at 80 °C. Finally, the metamaterial unit cell is fabricated upon these Mg_20,_ Mg_40,_ Mg_60,_ and Mg_80_ flexible substrates by copper sputtering.

### Fabrication of metamaterial on Mg–Zn ferrite based flexible composites

**Figure 3:**
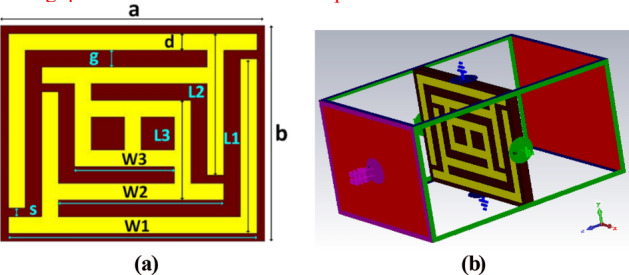
(**a**) The geometry and (**b**) the boundary setup of the metamaterial unit cell.

In this design, four different types of flexible substrates are used, termed Mg_20,_ Mg_40,_ Mg_60,_ and Mg_80,_ based on magnesium zinc ferrites (MgZnFe_2_O_4_). Figure 3a represents the geometrical view of the proposed unit cell. During CST simulation frequency-domain solver is used. The electromagnetic wave propagates from the positive z-axis to the negative z-axis, which is energized by two waveguide ports as the simulation setup is shown in Fig. 3b. The x and y boundaries are set as perfect electric (PEC) and perfect magnetic (PMC) walls. The necessary design parameters, along with their dimensions, are shown in Table 1. The substrate length is chosen 8 mm, and width *b* is chosen 6.5 mm, where the thickness of the substrate is 0.5 mm for all cases. The unit cell is fabricated on flexible substrates using copper sputtering with a thickness of 0.035 mm. Here, *L1* and *W1* are the length and width of the outer split ring resonator with a dimension of 5.25 mm and 7.5 mm, respectively. On the other hand, *L2* and *W2* are the length and width of the intermediate split ring resonator with a dimension of 4 mm and 5 mm, respectively. Moreover, *L3* and *W3* are the length and width of the inner split ring resonator with a dimension of 3 mm. A 0.25 mm gap is maintained among the edges of the substrate and the outer resonators, as well as 0.5 mm of gap *g* among the inner, middle, and outer resonators from all ends. The width of the copper strips *d* is also chosen 0.5 mm for conductive elements. Finally, a 0.25 mm gap *s* is maintained for all the splits.

**Table 1:** Design parameters of metamaterial unit cells.

Design parameter	Dimension	Design parameter	Dimension
	mm		mm
*a*	8.00	*W1*	7.50
*b*	6.50	*W2*	5.00
*L1*	5.25	*W3*	3.00
*L2*	4.00	*d = g*	0.5
*L3*	3.00	*s*	0.25

## Results and discussion

### Structure

**Figure 4:**
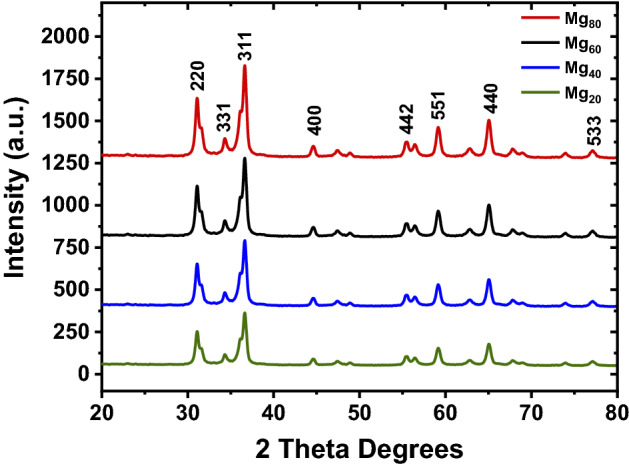
XRD patterns of the prepared Mg–Zn ferrites samples.

The Siemens D500 X-ray diffractometer having a Cu Kα anode (40 kV, 20 mA) with a 2θ angle range of 20° to 80° is used to confirm the phase formation of MgZnFe_2_O_4,_ and the recorded XRD plot for the synthesized samples are shown in Fig. 4.

The spinel structure of MgZnFe_2_O_4_ is identified and indexed with a principal peak of (311) for all Mg_20_, Mg_40,_ Mg_60_, and Mg_80_, respectively. The overall reflection peaks indexed at 2θ = 32° (220), 35° (331), 37° (311), 44° (400), 55° (442), 58° (551), 65° (440), 77° (533). Scherrer’s equation ^50^ as follows is used to calculate the average crystallite size of MgZnFe_2_O_4_ from the line width of the (311) reflection.

1 $$D = \frac{0.94 \lambda }{{\beta cos\theta }}$$

where λ is the wavelength of the X-ray radiation (1.54060 nm), and β is the full width at half maxima values in radians, and θ is the diffraction angle corresponding to the most intense reflection plane (311). The crystallite sizes of MgZnFe_2_O_4_ nano spinel that evaluated from the above equation (5) are 27.45 nm for Mg_20_, 25.75 nm for Mg_40_, 23.84 nm for Mg_60,_ 22.17 nm for Mg_80_, which is very close to the tabulated values reported by S. B. Somvanshi et al.^51^. The lattice constraint (*a*) was calculated with the help of Miller indices (*h k l*) information and interplanar spacing (*d*) values corresponding to leading peak (311) by the following equation:

2 $$a = d\sqrt {h^{2} + k^{2} + l^{2} } ,$$

**Figure 5:**
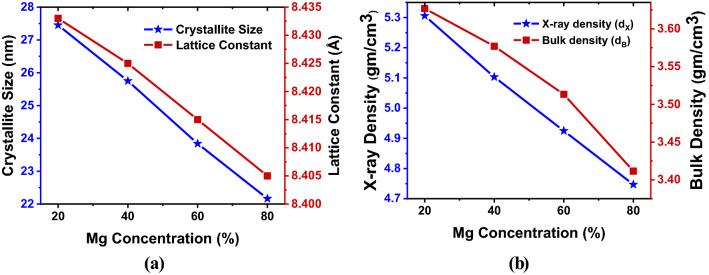
Variation (**a**) crystallite size and lattice constant and (**b**) X-ray density and bulk density of the prepared MgZnFe_2_O_4_ samples with different Mg concentrations.

The evaluated values of the lattice constraint of MgZnFe_2_O_4_ are *a* = 8.433 Å for Mg_20,_ *a* = 8.425 Å for Mg_40_, *a* = 8.415 Å for Mg_60_, and *a* = 8.405 Å for Mg_80,_ which also very similar to that presented in^51^. The calculated values of lattice constant (*a*) and average crystallite size (*D*) are accommodated in Table 2, and the compositional variation of lattice constant and average crystallite size concerning Mg^2+^ substitution is shown in Fig. 5a.

**Table 2:** Values of Lattice parameter (*a*), Unit cell volume (*V*), Average crystallite size (*D*), X-ray density (*d*_*x*_), Bulk density (*d*_*B*_), Porosity (*P*) for prepared Mg_x_Zn_(1−x)_Fe_2_O_4_ samples.

Mg concentration (%)	*a* (Å)	*V* (Å^3^)	*D* (nm)	*d*_*x*_ (gm/cm^3^)	*d*_*B*_ (gm/cm^3^)	*P* (%)
20	8.433	599.72	27.45	5.306	3.627	31.64
40	8.425	598.01	25.75	5.103	3.577	29.91
60	8.415	595.88	23.84	4.925	3.513	28.66
80	8.405	593.76	22.17	4.747	3.412	28.13

From Fig. 5a, it is seen that due to the increasing percentages of Mg^2+^ content x, the values of the lattice constant (*a*) and crystalline size are decreasing. This scenario has happened by following Vegard’s law^52^. Also, these decreasing characteristics can be validated based on theistinction of the ionic length of magnesium and zinc ions.

The volume (*V*) of the unit cell of the synthesized samples can be computed with the help of lattice constant (*a*) by the following formula:

3 $$V = a^{3}$$

As the unit cell volume values are directly proportional to the lattice parameter values, thus it shows the same trend as the values of the lattice constant. Hence, the X-ray density factor calculation is necessary to characterize materials. Therefore, the values of the X-ray density (*d*_*X*_) can be determined from the following equation:

4 $$d_{X} = \frac{Z \times M}{{V \times N_{A} }}$$

where *Z* represents the cubic lattice coordination number, *M* represents the molecular weight of individual concentrations, *V* is the volume of the unit cell, and *N*_*A*_ is the Avogadro’s number (i.e., *N*_*A*_ = 6.022 × 10^23^). Figure 5b represents the X-ray density (*d*_*x*_) and the bulk density (*d*_*B*_) variation with various compositions. It is investigated that the values of the X-ray density (*d*_*x*_) and the bulk density (*d*_*B*_) are decreases with an increase of Mg^2+^ ions. It may happen due to the molecular weight loss of the synthesized samples. Finally, from the values of the X-ray density (*d*_*x*_) and the bulk density (*d*_*B*_), the percentage of porosity (*P*) is calculated by the equation below:

5 $$P = 1 - \frac{{d_{B} }}{{d_{X} }}$$

The calculated values of the X-ray density (*d*_*x*_) and the bulk density (*d*_*B*_), and the percentage of porosity (*P*) are also summarized in Table 2.

### Morphology

**Figure 6:**
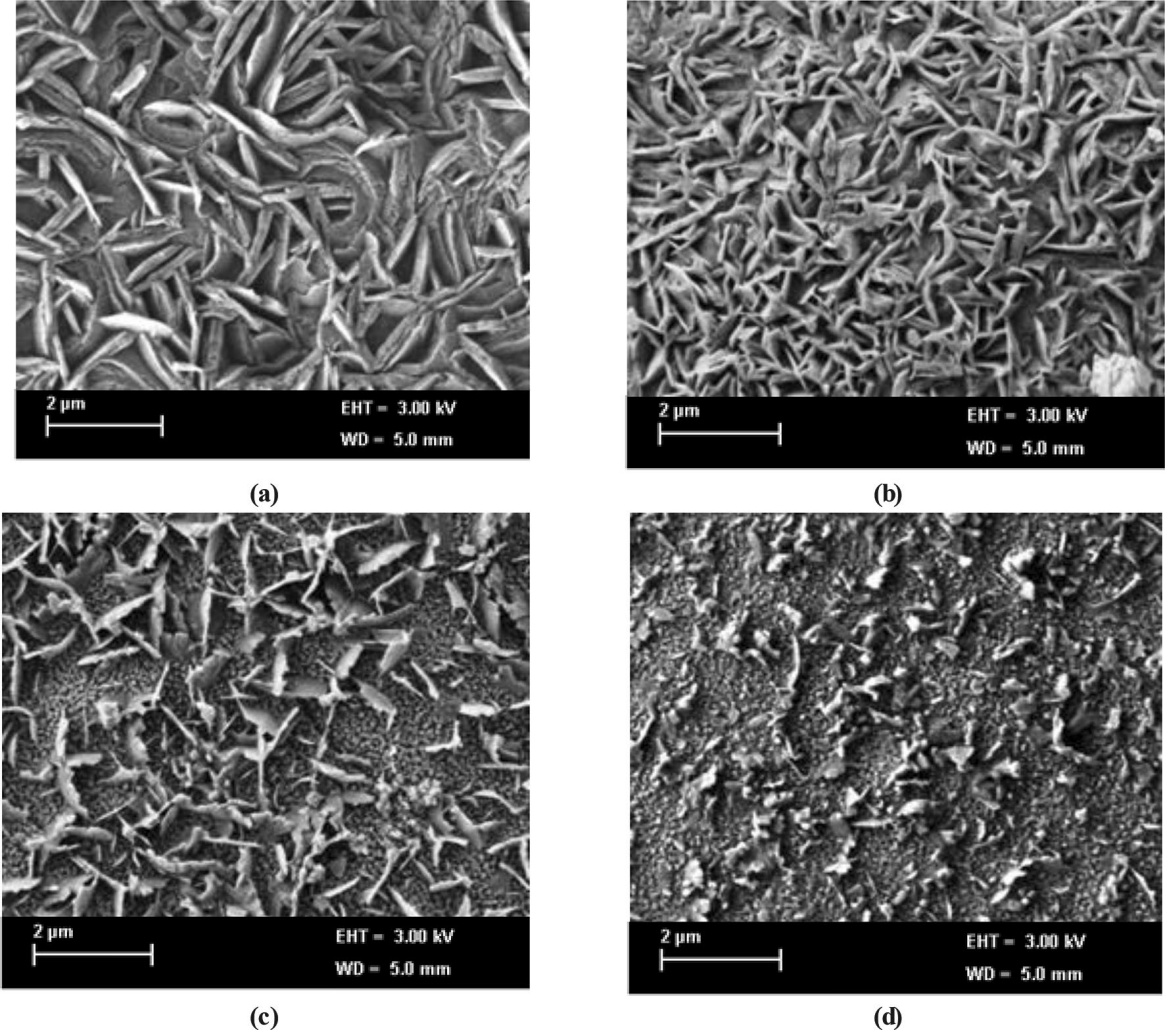
FSEM images of Mg–Zn ferrite nano powder (**a**) Mg_20_, (**b**) Mg_40_, (**c**) Mg_60,_ and (**d**) Mg_80_ respectively.

**Figure 7:**
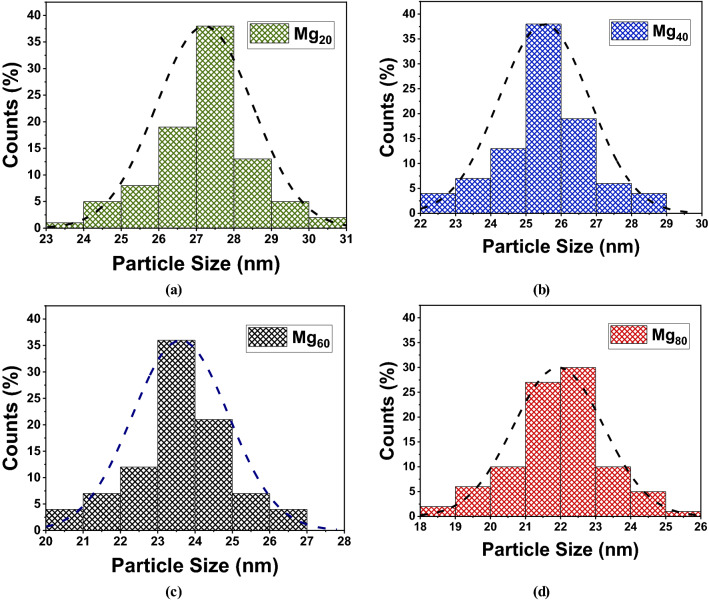
Particle size histogram of Mg–Zn ferrite nanopowder (**a**) Mg_20_, (**b**) Mg_40_, (**c**) Mg_60,_ and (**d**) Mg_80_ respectively.

The FESEM (Field Emission Scanning Electron Microscopy) technique is used to investigate the morphology of the synthesized nanoparticle. Figures 6 and 7 represents the FESEM images and particle size histograms of Mg_20_, Mg_40_, Mg_60_, and Mg_80._ A high-energy ball mill EMAX (Retsch, Germany) was utilized for grinding the Mg_x_Zn_(1−x)_Fe_2_O_4_ species to nanocrystals which allows faster grinding with a maximum revolutionary speed of 2000 rpm and produce unique size particles. Zirconium oxide grinding balls having 0.5 mm size were used to grind the materials for about 3 hours. The mean grain size of the synthesized nanoparticle is approximately 27.30 nm for Mg_20_, 25.60 nm for Mg_40_, 23.70 nm for Mg_60,_ and 22.02 nm for Mg_80_, which have a good agreement with the values obtained from XRD analysis. It is also observed that with increases in Mg content, the grain size and porosity are decreased, which also significantly affects on dielectric properties of the materials and leads to having tunable properties.

#### Optical and photoluminescence analysis

**Figure 8:**
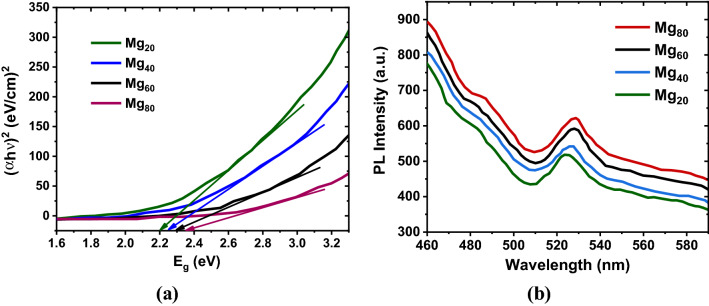
(**a**) Tauc plots and (**b**) PL spectra of Mg–Zn ferrite samples.

The optical energy bandgap (E_g_) of the prepared samples is determined using the UV–Vis spectrophotometer. The wavelength of the applied light is chosen from 300 nm to 800 nm. For evaluating the optical energy bandgap (E_g_) values, the most popular “Tauc plot” [(αhν)^2^ v/s (E_g_)] was drawn from the UV–Vis absorbance spectral data. The “Tauc plot” was drawn using the following relation, and the plots are displayed in Fig. 8a.

6 $$\alpha = \frac{{A(h\upsilon - E_{g} )^{n/2} }}{h\upsilon }$$

The optical bandgap energy (E_g_) parameters were determined from the tangent drawn at the X-axis of the “Tauc plots,” as shown in Fig. 8a. The values of ‘E_g_’ were found to be in the range of 2.20–2.36 eV. The photo-luminescent properties were studied for the prepared samples by the photoluminescence (PL) spectra excited at a wavelength of 400 nm. Fig. 8b demonstrates the PL spectra recorded at 300 K. The distinctive near band-edge emission (NBE) for all the samples was observed at the wavelength range of 524–530 nm. These variations in optical energy bandgap (E_g_) and PL spectra is happened due to variations of material concentration as well as grain size, cation distribution, etc.

#### Magnetic analysis

**Figure 9:**
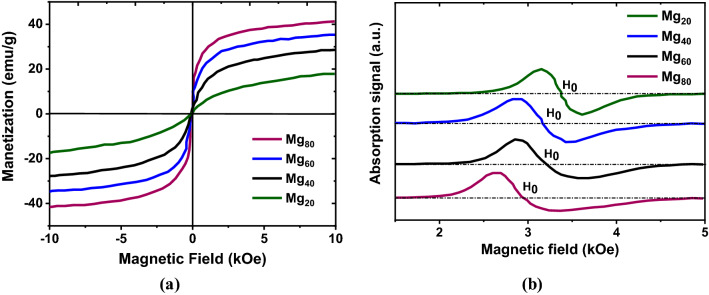
(**a**) Comparison of magnetization versus magnetic field (M–H) hysteresis loops and (**b**) ferromagnetic resonance (FMR) spectra of Mg–Zn ferrite samples.

The M–H hysteresis loops from the prepared Mg–Zn ferrite samples are presented in Fig 9a, where M and H stand for the magnetization and magnetic field values, respectively. The M–H hysteresis data are collected by a SQUID-VSM magnetometer at room temperature, and very narrow loops are observed having almost no hysteresis behavior. Thus, the prepared Mg–Zn ferrite samples exhibit the properties like soft magnetic material. Figure 9a also shows that the Mg_x_Zn_(1−x)_Fe_2_O_4_ nanoparticles' maximum magnetization increased with increases in the Mg content. It is noted that the magnetic hysteresis loops of the Mg–Zn ferrite nanoparticles did not reach complete saturation even when the applied magnetic field was 10 kOe. This feature is often found in spinel ferrite nanoparticles. It can be ascribed to the presence of a spin-disordered layer on nanoparticle surfaces, which requires a large magnetic field to saturate together with the concomitant effect of the size of the ultrafine ferrite particles. The ferromagnetic resonance (FMR) spectra of four Mg–Zn ferrite nanoparticle samples are plotted in Fig. 9b. The resonance field was found to be between 2.65 and 3.20 kOe. The magnetic study revealed that the obtained Mg–Zn ferrite nanoparticles have a potential application in the microwave region.

#### Dielectric properties

**Figure 10:**
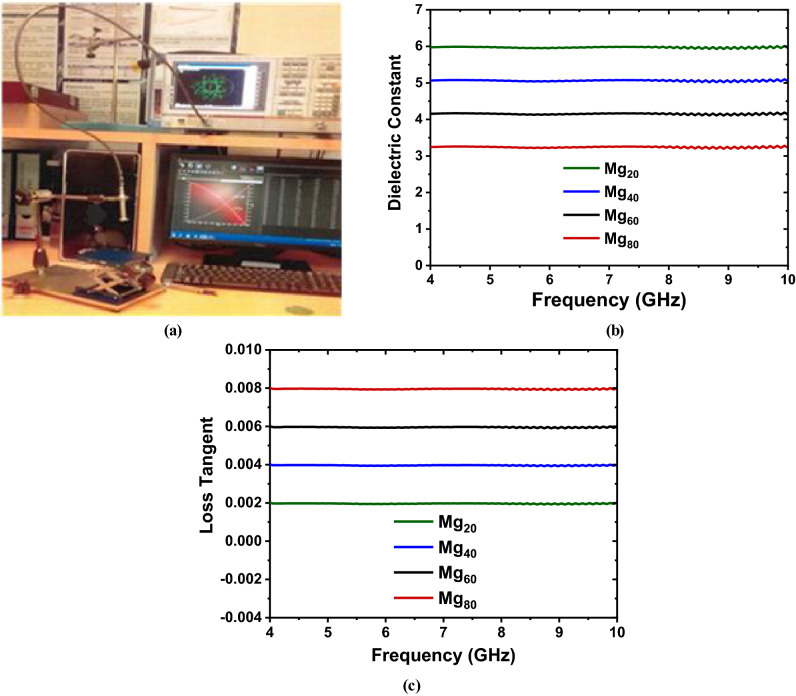
(**a**) Dielectric measurement setup with DAK 3.5 kit, (**b**) Dielectric constant, and **(c)** Loss tangent (T_δ_) over frequency.

The dielectric constants (${{\varvec{\varepsilon}}}_{{\varvec{r}}}$) and loss tangents (tan δ) of the prepared, flexible substrates is measured at a frequency range of 4-10 GHz with the DAK 3.5 (200 MHz to 20 GHz) dielectric assessment kit as shown in Fig. 10a, manufactured by Schmid and Partner AG, Switzerland, and the plot are shown in Fig. 10b and c respectively. With the increase of applied frequency on the specimen under test, the values of the dielectric constants (${\varepsilon }_{r}$) and loss tangents (tan δ) have slightly fluctuated, which is consistent with Koop’s phenomenological hypothesis and the Maxwell-Wagner model of interfacial polarization^53,54^. The calculated values of the relative permittivity (${{\varvec{\varepsilon}}}_{{\varvec{r}}}$) is 6.01 for Mg_20,_ 5.10 for Mg_40,_ 4.19 for Mg_60_ and 3.28 for Mg_80_, whereas the values of loss tangents (tan δ) are 0.002 for Mg_20_, 0.004 for Mg_40_, 0.006 for Mg_60_ and 0.008 for Mg_80_. The porosity and the value of the dielectric constants decrease from 6.01 (Mg_20_) to 3.28 (Mg_80_) with increases of Mg content, while the value of loss tangents (tan δ) is increased with increases of Mg content. This variation is originating from the grain size variations of synthesized nanoparticles as found in XRD and SEM analysis too. Thus, the dielectric properties of the prepared MgZnFe_2_O_4_ composites can be tuned by tuning the molar ratios, which is very effective where the predefined or arbitrary values of different dielectric properties are required. Finally, as the prepared, flexible substrates based on MgZnFe_2_O_4_ offers low values of the relative permittivity (${{\varvec{\varepsilon}}}_{{\varvec{r}}}<15$) and very low values of loss tangent (tan δ), it can be used as microwave dielectric material suitably.

#### Electromagnetic properties of the fabricated metamaterials

There has been a wide interest in the application of metamaterials in recent years. The materials in nature are made of atoms or molecules, while the metamaterial is engineered with artificially ordered repetitive structures. Those newly designed structures laid above the base material, including but not limited to their shape, arrangement, and geometry, dominate the property of the metamaterial. Those repetitive structures, often called periodic unit cells, are usually assembled at a scale below the wavelength to manipulate the electromagnetic property of the wave. The property of a metamaterial depends on the structure of the unit cell, equivalent to the atom or molecule of natural materials. The performance of metamaterial could vary upon the modification of those unit cells. In contrast, metamaterial may be designed to manipulate numerous properties of those applications influenced by the electromagnetic wave. Here, CST microwave studio is used to finalize the structure of the metamaterial unit cell before prototyping. Finally, the electromagnetic properties of the proposed flexible metamaterial were measured and extracted with the PNA vector network analyzer (Agilent N5227A, 10 MHz-67 GHz).

**Figure 11:**
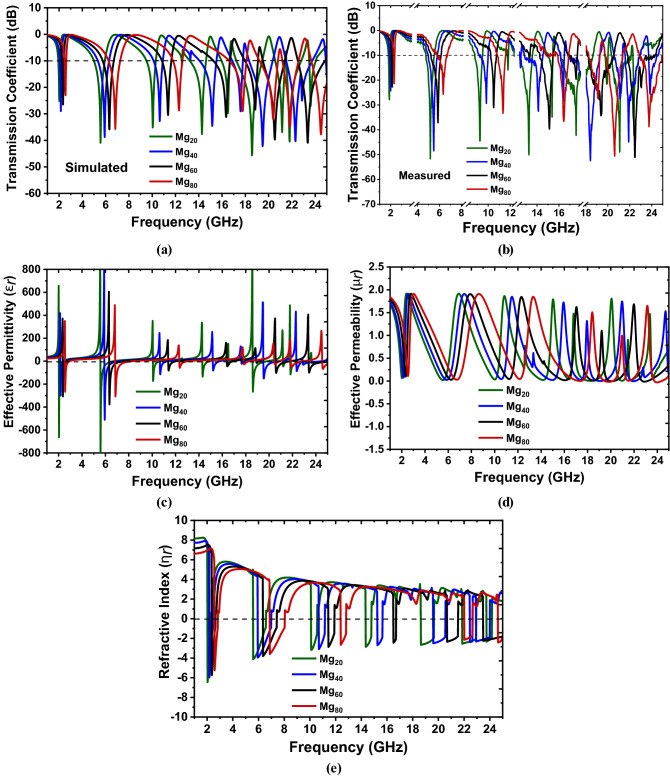
The amplitude of (**a**) the simulated transmission coefficient, (**b**) the measured transmission coefficient, the extracted effective (**c**) permittivity, (**d**) permeability, and (**e**) refractive index for all four concentration of magnesium Mg_20_, Mg_40_, Mg_60_, and Mg_80_.

The simulated transmission coefficient (S_21_) for the proposed metamaterial unit cell on all the four flexible substrates, i.e., Mg_20,_ Mg_40,_ Mg_60,_ and Mg_80,_ are shown in Fig. 11a, and the corresponding measured transmission coefficient are shown in Fig. 11b. The effective permittivity, permeability, and refractive index extracted by the Nicolson-Ross-Wire method^55^ are presented in Fig. 11c,d, and e, respectively. Based on the Nicolson-Ross-Wire method, the values of the effective permittivity ($\varepsilon_{r}$), permeability ($\mu_{r}$), and refractive index ($n_{r}$) can be calculated by the following equations:

7 $$\varepsilon_{r} \sim \frac{2}{{jk_{0} d}} \times \frac{{\left( {1 - V_{1} } \right)}}{{\left( {1 + V_{1} } \right)}}$$

8 $$\mu_{r} \sim \frac{2}{{jk_{0} d}} \times \frac{{\left( {1 - V_{2} } \right)}}{{\left( {1 + V_{2} } \right)}}$$

9 $$n_{r} = \sqrt {\mu_{r} \varepsilon_{r} }$$

where $k_{0} = \frac{2\pi f}{c}$, $c$ is the speed of light, and $d$ is the thickness of the substrate. Also, $V_{1} = |S_{11} | + |S_{21} |$ and $V_{2} = |S_{21} | - |S_{11} |$ in which S_11_ and S_21_ are the reflection and transmission coefficient, respectively.

In the case of Mg_20,_ there are nine simulated resonances observed tabulated in Table 5, whereas there are ten resonances were found during measurement by Mg_20._ With Mg_40,_ there are seven simulated resonances observed, whereas there are nine resonances were found during measurement by Mg_40_. For both Mg_60_ and Mg_80,_ there are seven simulated, and measured resonances were observed, and all of these are tabulated in Table 5. A very good agreement is found among simulated and measured values with a slight frequency shifting. This frequency shifting may happen due to fabrication error and/or external noise during measurements. All the values of effective permeability (*μ*_*r*_) were found positive, and the values of effective permittivity (*ε*_*r*_) were found negative, corresponding to resonance frequencies. Thus the metamaterial can be declared as single negative (SNG) or epsilon negative (ENG) metamaterial. The negative values of the effective permittivity and refractive index are presented in Table 3. The parameters are quite similar in the pattern for all these four metamaterials but at slightly different frequencies due to having slightly different dielectric properties.

**Table 3:** Negative values of effective parameters among Mg_20_, Mg_40_, Mg_60_, and Mg_80_.

Mg concentration	Effective parameters	Negative frequency region (GHz)
Mg_20_	Permittivity (*ε*_*r*_)	2.02–2.36, 5.57–6.74, 10.06–10.70, 14.29–14.91, 16.53–16.73, 18.59–19.87, 21.18–21.43, 21.81–23.28, 21.64–25
	Refractive Index (*n*_*r*_)	2.02–2.32, 5.57–6.58, 10.09–10.57, 14.33–14.79, 18.63–19.66, 21.85–22.95, 23.74–24.17
Mg_40_	Permittivity (*ε*_*r*_)	2.14–2.51, 5.91–7.13, 10.66–11.30, 15.18–15.79, 17.63–17.89, 19.51–20.77, 22.33–22.78, 22.87–24.57
	Refractive Index (*n*_*r*_)	2.16–2.45, 5.93–6.99, 10.68–11.17, 15.24–15.66, 19.58–20.55, 22.44–22.66, 22.92–24.01
Mg_60_	Permittivity (*ε*_*r*_)	2.36–2.73, 6.32–7.65, 11.38–12.09, 16.38–18.92, 18.89–19.14, 20.55–21.82, 23.38–25
	Refractive Index (*n*_*r*_)	2.36–2.65, 6.34–7.42, 11.42–11.91, 16.49–16.71, 20.67–21.53, 23.47–25
Mg_80_	Permittivity (*ε*_*r*_)	2.54–2.95, 6.84–8.33, 12.31–13.07, 17.81–18.30, 20.45–20.88, 21.86–22.98, 24.54–25
	Refractive Index (*n*_*r*_)	2.57–2.84, 6.86–8.05, 12.38–12.82, 22.01–22.66, 24.62–25

**Figure 12:**
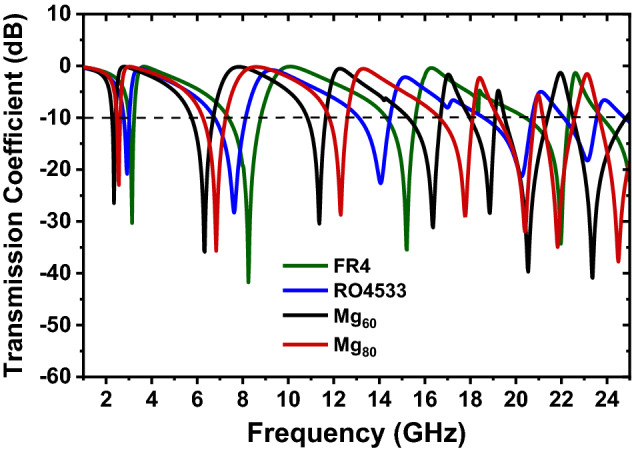
Comparison of transmission coefficients among proposed material with conventional materials.

To justify the nobility of proposed flexible substrate materials, the transmission of proposed metamaterials was investigated by replacing the substrate materials with conventional FR4 and Rogers RO4533 materials keeping the unit cell structure remain the same. The obtained transmission coefficient regarding the above cases is shown in Fig. 12. The dielectric constant (***ε***_***r***_) values of FR4 and Mg_60_ are similar but different in loss tangent values. For the same size and structure of the unit cell, there are only five resonances observed with FR4. Besides, with Mg_60,_ there are seven resonances observed and covers more bands of microwave regime. On the other hand, also the dielectric constant (***ε***_***r***_) values of RO4533 and Mg_80_ are almost similar but different in loss tangent values, and only five resonances are found with RO4533, whereas there are seven resonances are investigated with Mg_80_. All the prepared, flexible substrates, i.e., Mg_20,_ Mg_40,_ Mg_60,_ and Mg_80_ cover S-, C-, X-, Ku-, and K-band of the microwave regime. Some of the related comparisons among proposed flexible substrates Mg_60_ and Mg_80_ with FR4 and RO4533 in terms of substrate type, material type, permittivity, loss tangent, number of resonances, and the band of applications are presented in Table 4. It is seen that in both cases, the proposed materials offer better performances over commercially available materials, which originates due to having tunable dielectric and magnetic properties. The dielectric and magnetic properties are varied for the variations of material percentages, and the percentage of the materials changes the metal and insulator interface of the compounds. And the values of the micro-capacitors array that formed in between the metal-insulator surface also become change, affecting the overall performances. Moreover, the proposed substrates materials are highly flexible, lightweight, and low in cost compared to other commercially available materials.

**Table 4:** Comparison of proposed materials with conventional substrate materials.

Substrate type	FR4	RO4533	Mg_60_	Mg_80_
Material type	Hard	Hard	Flexible	Flexible
Permittivity (*ε*_*r*_)	4.30	3.45	4.30	3.40
Loss tangent (T_δ_)	0.025	0.0025	0.006	0.008
Number of resonances	5	4	7	7
Band of application	S, C, Ku, and K	S, X, Ku, and K	S, C, X, Ku, and K	S, C, X, Ku, and K

A summary of the fabricated flexible metamaterials based on sol-gel synthesized Mg–Zn ferrite [Mg_x_Zn_(1−x)_Fe_2_O_4_] composites for all four concentration of magnesium, i.e., Mg_,_ Mg_40,_ Mg_60,_ and Mg_80,_ is presented in Table 5 in terms of total dimension, substrate material, operating frequency, resonances, metamaterial type and microwave band of applications. Also, a brief comparison of the proposed metamaterial unit cells in terms of (1) physical dimensions, (2) the type of substrate, (3) the number of resonances obtained, (4) the band of applications, (5) the effective medium ratio (EMR), and (6) the type of metamaterials with some existing literature is presented in Table 6. Here, the EMR is an important factor regarding metamaterials design, which governs the compactness of the designed metamaterial. The EMR is calculated by the following equation.

10 $$EMR = \frac{{Wavelength\;of\;the\;unit\;cell\;\left( \lambda \right)}}{{Length\;of\;the\;unit\;cell\;\left( L \right)}}$$

**Table 5:** Summary of the designed metamaterials on flexible composites.

Properties	Metamaterial on flexible composites			
	With Mg_20_	With Mg_40_	With Mg_60_	With Mg_80_
Total dimension	8 × 6.5 mm^2^	8 × 6.5 mm^2^	8 × 6.5 mm^2^	8 × 6.5 mm^2^
Electric dimension	0.054 λ × 0.044 λ	0.057 λ × 0.047 λ	0.063 λ × 0.051 λ	0.068 λ × 0.055 λ
Substrate material	Magnesium zinc ferrite with a composition of (Mg_0.2_Zn_0.8_Fe_2_O_4_)	Magnesium zinc ferrite with a composition of (Mg_0.4_Zn_0.6_Fe_2_O_4_)	Magnesium zinc ferrite with a composition of (Mg_0.6_Zn_0.4_Fe_2_O_4_)	Magnesium zinc ferrite with a composition of (Mg_0.8_Zn_0.2_Fe_2_O_4_)
Operating frequency	1.93–24.07 GHz	2.06–23.74 GHz	2.27–24.86 GHz	2.46–25^+^ GHz
Simulated resonance frequency	2.03 GHz, 5.56 GHz, 10.05 GHz, 14.27 GHz, 16.50 GHz, 18.57 GHz, 21.16 GHz, 21.81 GHz, and 23.61 GHz	2.15 GHz, 5.92 GHz, 10.65 GHz, 15.17 GHz, 17.58 GHz, 19.48 GHz, and 22.31 GHz	2.35 GHz, 6.33 GHz, 11.34 GHz, 16.36 GHz, 18.86 GHz, 20.54 GHz, and 23.37 GHz.	2.56 GHz, 6.83 GHz, 12.31 GHz, 17.77 GHz, 20.40 GHz, 21.84 GHz, and 24.50 GHz
Measured resonance frequency	1.89 GHz, 5.18 GHz, 9.27 GHz, 11.80 GHz, 13.26 GHz, 15.38 GHz, 17.56 GHz, 19.94 GHz, 21.09 GHz, and 22.38 GHz	2.01 GHz, 5.49 GHz, 9.83 GHz, 14.13 GHz, 16.41 GHz, 18.43 GHz, 21.14 GHz, 21.90 GHz, and 22.31 GHz	2.15 GHz, 5.87 GHz, 10.51 GHz, 15.14 GHz, 17.58 GHz, 19.38 GHz, and 22.48 GHz.	2.32 GHz, 6.35 GHz, 11.35 GHz, 16.43 GHz, 19.10 GHz, 20.63 GHz, and 23.73 GHz
EMR	18.47	17.44	15.96	14.65
Applications	S-, C-, X-, Ku-, and K-Band	S-, C-, X-, Ku-, and K-Band	S-, C-, X-, Ku-, and K-Band	S-, C-, X-, Ku-, and K-Band

**Table 6:** Comparison of proposed metamaterial with some existing literature.

References	Dimensions (mm)	Substrate type	No. of Resonances	Band	EMR	Observations
^24^	25 × 20	Flexible	7	S-, C-, X	3.75	Larger dimension, narrower bandwidth, and low EMR
^25^	12.5 × 10	Flexible	2	X-, Ku	2.88	Dual-band only, and very poor EMR value
^40^	10 × 8	Hard	1	X	3.40	Polarization-dependent, non-flexible, and low EMR
^41^	9 × 9	Hard	3	C-, X-, Ku	5.00	Non-flexible with poor EMR value
^42^	12 × 12	Hard	2	S-, C	5.55	Non-flexible, dual-band only with poor EMR value
Proposed	8 × 6.5	Flexible	7–9	S-, C-, X-, Ku-, and K	14.65–18.47	Highly flexible, lightweight, low electrical dimension, improved EMR, and polarization-independent

where λ is the electrical wavelength corresponding to the lowest resonance of the transmission parameter (S_21_) obtained by the metamaterial unit cell and L is the maximum physical length of the metamaterial unit cell. The expected value of EMR is >4 to achieve the negative permittivity and/or permeability as well as the negative refractive index to perform as a metamaterial. From Table 6, it is observed that Rahman et al. in 2018, have prepared NiAl2O4 based flexible substrates for metamaterials that offered negative electromagnetic properties with a quite large dimension of 25×20 mm2 and applicable for S-,C-, and X-bands of microwave regime having seven resonances with very narrow bandwidth at each resonance with an EMR value of 3.75 only^24^. In 2019, a double negative metamaterial on flexible nickel aluminate substrate was proposed by Faruque et al. with a dimension of 12.5×10 mm2 having dual-band (X and Ku) only with a very poor EMR value of 2.88^25^. A metamaterial having tunneled structure was demonstrated by Ahmed et al. in 2019 with a dimension of 10×8 mm2, but it is polarization-dependent and has no flexibility as fabricated on conventional hard FR4 substrate. This metamaterial offered only a single microwave band (X) of application, and the value of EMR is 3.40 only^40^. Hasan et al. in 2017, presented a tri-band meta atom on hard Rogers RT 5880 substrate with a reduced dimension of 9×9 mm2 for having an EMR value of 5 only^41^. Overall, it is seen that the proposed flexible metamaterial is electrically compact and possessed with improved EMR value, wide bandwidth, and SNG properties. Thus, the proposed metamaterials with new flexible microwave substrates overcome all the previous significant drawbacks like low EMR, narrow bandwidth, larger size, etc. and it is suitably applicable for S-, C-, X-, Ku-, and K-band of microwave regime as well as within flexible microwave technology.

## Conclusion

In this study, the authors have been developed SNG metamaterial upon sol-gel synthesized Mg_x_Zn_(1−x)_Fe_2_O_4_ based flexible microwave composites having four different compound ratios termed Mg_20,_ Mg_40,_ Mg_60,_ and Mg_80_. Due to having different compositional ratios, they possess various/tunable structural and microwave properties. The synthesized composites have average crystallite sizes of the spinel from 20 to 24 nm, and they exhibit high dielectric permittivity values that varied from 6.01 to 3.28 and loss tangents from 0.002 and 0.008, for Mg_20_ to Mg_80,_ respectively. Moreover, the fabricated metamaterials on flexible composites offer a wide band of operating frequencies with single negative characteristics at nine different resonances by Mg_20_ and at seven different resonances by the rest of all (Mg_40,_ Mg_60_, and Mg_80_) that ranges from S- to K-band of microwave regime. A very good agreement is found among simulation and measured values with improved EMR values that are calculated from 14.65 to 18.47. The advantages of the prepared composites are they are cost-effective, highly flexible, lightweight, and applicable in the case of wearable devices, and also have shown better performances comparing with conventional FR4 and Rogers RO4533 substrates. Thus, the overall investigations confirm that the proposed flexible metamaterials are the prominent candidate for the S-, C-, X-, Ku- and K-bands of the microwave frequency range as well as flexible microwave technologies.
